# Remarks on the Use of Prussic Acid in Affections of the Stomach and Heart

**Published:** 1823-12

**Authors:** R. Macleod


### Art. VII.—*Remarks on the Use of Prussic Acid in Affections of the Stomach and Heart.* By R. Macleod, M.D. &c.

In a communication made to this Journal about two years ago, I had occasion to allude to the good effects which appeared to me to arise from the use of prussic acid in certain kinds of dyspepsia. More extended observation has confirmed me in regarding this medicine as a remedy of considerable power in those forms of indigestion which are attended with much pain of the stomach and flatulence. It is extremely difficult to judge accurately of the powers of a medicine of this kind; and I would not, therefore, be understood as bestowing upon it unqualified praise, as universal in its application, or invariable in its effects. At the same time, I am inclined to think it merits a more extensive trial in this class of diseases, than, so far as I know, it has hitherto obtained.

One of the most distressing symptoms attending dyspepsia is uneasiness in the chest, with occasional fits of palpitation, sometimes assuming the more severe character of angina pectoris. On the other hand, in primary organic diseases of the heart, we very frequently find many of the most troublesome symptoms of dyspepsia, particularly flatulence; and such cases, although not altogether dependent on the state of the stomach, are nevertheless much aggravated by its derangement. Now, in such instances of morbid action of the heart brought on by dyspepsia, or of dyspepsia sympathetic of organic disease of the heart, I am inclined to think that much benefit is to be obtained from the employment of prussic acid. That, in the latter class of cases, it can for a moment retard the progress of organic mischief, I would by no means wish to imply, but, after a fair trial, I have been led to believe that it may palliate the sufferings of the patient, although it cannot cure the disease. In a case to which I alluded on a former occasion, the relief afforded was of the most unequivocal kind, although other remedies had failed to produce even temporary benefit. This patient died soon after, and the heart was found to be very considerably enlarged, and extremely soft.

A short time after the occurrence of this case, a patient applied to me, who laboured under well-marked symptoms of enlargement of the heart, attended with considerable pain and palpitation of this organ. He was dyspeptic. I prescribed ten drops of prussic acid in a five-ounce mixture, of which he began by taking two table-spoonsful three times a-day, gradually increased till he took the whole of the mixture in twenty-four hours. After a few days the symptoms began to yield, and he became much more comfortable. Having persevered in his medicine for about a fortnight, he discontinued his attendance, and I saw nothing of him for several months. The only perceptible effect from the medicine was the improvement in his general state of health, and diminution of the violence of the action of the heart: he was less subject to fits of palpitation, but I could not perceive that, in the intervals, the number of pulsations (averaging from seventy-five to eighty-five,) was diminished. After some months, this patient returned with symptoms analogous to those above described, and, without waiting to be examined, asked for *the medicine:* he took it a second time, with similar benefit. From this period, the intervals during which he was able to omit the use of the prussic acid gradually diminished in proportion as the organic derangement increased: notwithstanding this, however, it continued to afford relief until within a few weeks of his death, which did not take place for two years from the time of his falling under my notice. He had tried a variety of medicines, without obtaining any benefit, before he had recourse to the prussic acid; and, after this failed to afford relief, no other remedy was of the slightest avail. An opportunity presented itself of examining the body of this patient, who was under thirty-five at the time of his death: the only appearances worthy of note were great enlargement of the heart, with ossification of the mitral valves, and of those at the root of the aorta.

This is the second case in which I have seen prussic acid afford relief in derangement of the functions of the heart, which has been proved by dissection to have originated in organic disease. I have likewise given it in many cases of palpitation, and other unpleasant symptoms referred to the region of the heart, where these have appeared to depend on dyspepsia, and generally with considerable advantage. I am decidedly of opinion that it gives most relief where there is evidence of the disease in the chest, whether primary or secondary, being connected with faulty digestion: but I do not regard this as its only *modus operandi*, because I have not seen the same benefit derived when the dyspepsia has been as entirely removed by other means.

Different opinions may be entertained with respect to the evidence advanced in favour of this medicine, either here or elsewhere, and there is, of course, fair grounds for a certain degree of scepticism; but there is one point with regard to which I am convinced many are in error. I mean the degree of danger attending the use of prussic acid. I am in the habit of giving, as a general prescription, ten drops in five ounces, either of distilled or of peppermint water, of which mixture adults (I do not use it for children,) begin with two tablespoonsful three times a-day, gradually increased; and from this I have very seldom seen any inconvenience, and never any that was serious. The largest dose I have ever given is twenty-eight drops in twenty-four hours: Dr. HELLER* mentions that he has pushed it to the extent of sixty drops of the *medicated*, —that is to say, about fifteen of the *pure*, prussic acid in a day.

*Henrietta-street; Nov. 10th,* 1823.

---

* *Revue Medicale*, Aout.

